# Continuous electroencephalography in the medico-surgical intensive care setting in Brazil: initial experience after 4 months of implementation

**DOI:** 10.1186/cc9753

**Published:** 2011-03-11

**Authors:** P Kurtz, D Santos, P Horta Gomes, C Andre, R Lima, J Kezen, L Lopes, M Kalichsztein, G Nobre

**Affiliations:** 1 Casa de Saúde São José, Rio de Janeiro, Brazil; 2 Hospital Samaritano, Rio de Janeiro, Brazil

## Introduction

The objective of this study was to analyze the prevalence, risk factors and impact on outcome of electrographic seizures (ESz), nonconvulsive status epilepticus (NCSE), and periodic epileptiform discharges (PEDs) in critically ill patients admitted to two mixed medico-surgical ICUs.

## Methods

This was a retrospective study of 58 consecutive ICU patients (mean age 68 ± 23 years old; 50% women) who underwent continuous electroencephalography (cEEG) monitoring for altered mental status. Outcome was assessed as hospital mortality.

## Results

Sixteen patients (28%) were admitted with a primary neurological diagnosis. Mean duration of cEEG was 12 ± 17 hours. Thirty-four patients (59%) were comatose and 32 patients were mechanically ventilated (55%) during cEEG monitoring. Seventeen percent (*n *= 10) had ESz, 10% (*n *= 6) had NCSE, 19% (*n *= 11) had periodic lateralized epileptiform discharges and 26% (*n *= 15) had epileptiform discharges.

## Conclusions

In a mixed population of medical and surgical patients, ESz and NCSE are frequent and associated with increased hospital mortality.

